# Galectin-3 and β-trace protein concentrations are higher in clinically unaffected patients with Fabry disease

**DOI:** 10.1038/s41598-019-42727-4

**Published:** 2019-04-17

**Authors:** Diana Hernández-Romero, Jessica Sánchez-Quiñones, Juan Antonio Vílchez, José Miguel Rivera-Caravaca, Gonzalo de la Morena, Gregory Y. H. Lip, Vicente Climent, Francisco Marín

**Affiliations:** 1 Department of Cardiology, Hospital Clínico Universitario Virgen de la Arrixaca, Instituto Murciano de Investigación Biosanitaria (IMIB-Arrixaca), University of Murcia, CIBERCV, Murcia, Spain; 2 Department of Cardiology, Hospital General Universitario de Alicante, Alicante, Spain; 3 Department of Clinical Analysis, Hospital General Universitario Santa Lucía, Instituto Murciano de Investigación Biosanitaria (IMIB-Arrixaca), Cartagena, Spain; 4 Liverpool Centre for Cardiovascular Science, University of Liverpool and Liverpool Heart & Chest Hospital, Liverpool, United Kingdom; 5 Aalborg Thrombosis Research Unit, Department of Clinical Medicine, Aalborg University, Aalborg, Denmark

**Keywords:** Diagnostic markers, Predictive markers, Prognostic markers, Cardiovascular diseases

## Abstract

Current therapies have not shown benefit in organ damage reversal in Fabry disease (FD), but biomarkers could help risk stratification and prognosis. We investigated if several biomarkers of cardiac fibrosis, cardiac wall stress, myocardial injury, renal function and inflammation, are associated with early cardiac affectation in FD patients. We included FD patients from four cardiology outpatient clinics of southeastern Spain. At inclusion, Galectin-3 (Gal-3), N-terminal proB-type natriuretic peptide, high sensitivity troponin T (hsTnT), β-trace protein (BTP) and interleukin-6 concentrations were measured. The relation of biomarkers concentrations with clinical features, cardiac involvement and organ affectation according to the Mainz Severity Score Index (MSSI) was investigated. 44 FD patients (n = 21 affected and n = 23 unaffected) were compared to age and sex-respectively matched healthy controls. Significant differences in biomarkers’ concentration between FD groups were observed. Importantly, Gal-3 and BTP levels were higher in unaffected patients when compared with age and sex-matched healthy controls (both p < 0.05). All the biomarkers correlated with clinical features. When cut-off values for clinical affectation (measured as MSSI ≥ 20) were established, only hsTnT (OR 30.69, 95% CI 2.70–348.42) and male sex (OR 8.17, 95% CI 1.16–57.75) were independently associated with cardiac damage by multivariate regression analysis. Gal-3 and BTP levels are increased in unaffected FD patients compared to healthy controls. This suggests that these biomarkers could be useful for the early detection of cardiac affectation in FD patients. On the other hand, hsTnT and male sex are independent risk factors for established clinical cardiac damage in FD.

## Introduction

Fabry disease (FD) is a rare inherited lysosomal storage disorder linked to the X chromosome. It is characterized by a non-metabolized glycosphingolipids progressive accumulation in several tissues. FD can affect many different organs showing neurological, cutaneous, ocular, cardiac, gastrointestinal and renal manifestations^1–3^. Published data suggest that heterozygote females present slower and milder progression and expression of the disease^4,5^ although there are reports showing similar severity than to hemizygous males^6,7^.

Due to the heterogeneity in clinical manifestations of patients with FD, the Mainz Severity Score Index (MSSI) was proposed as an attempt to develop a disease-specific scoring system^8^. This scheme covers different areas including cardiac signs and symptoms. Indeed, cardiac manifestations such as arrhythmias, chronic heart failure and small vessel disease occur frequently in patients with FD^9^. In addition, cardiac involvement presenting as left ventricular hypertrophy (LVH) caused by myocardial replacement fibrosis is typically observed in advanced stages of the disease^10^.

Nonetheless, organ and system involvement is progressive in FD, with most patients developing severe kidney and heart disease by the third to fifth decade of life^11^. Despite that the available evidence suggests that specific treatment using enzyme replacement therapy (ERT) with recombinant αGAL-A (agalsidase), is able to slow down the progression of FD, unfortunately further reversal of organ involvement seems to remain out of reach^12^. This fact supports the importance of future research on the identification of early cardiac damage.

Although several studies evaluating the usefulness of different biomarkers in FD and other cardiomyopathies^13–15^, no biomarker has proven to be useful in assessing underlying pathophysiological mechanisms, nor screen for (or predict) the disease development in FD for earlier initiation of ERT^16–18^.

Since FD is a progressive disease and current therapies have not shown benefit in organ damage reversal, biomarkers of early organ involvement could help risk stratification and help determine prognosis. In the present study, we have measured selected biomarkers of different molecular pathophysiological pathways, including cardiac fibrosis, cardiac wall stress, myocardial injury, renal function and inflammation, in order to investigate if any is associated with early cardiac affectation in FD patients.

## Material and Methods

We recruited consecutive patients with FD attending cardiology outpatient clinics in four hospitals of the southeastern Spain. The diagnosis was performed in all cases by genetic confirmation. We excluded patients with hepatic or renal failure (creatinine clearance <50 ml/min), and chronic inflammatory or neoplasic diseases. A complete history and clinical examination was performed, including 12-lead electrocardiogram (ECG), standard echocardiography, and, when available a blinded cardiac magnetic resonance study. All of these were acquired from medical history and electronic medical records. The transthoracic echocardiogram was performed using an iE33 ultrasound imager (Philips Medical System, Andover, Massachusetts, United States). For the measurement of the linear dimensions of the LV, two-dimensional images of the long axis obtained from a parasternal window were used. Regarding the RV, its systolic function was assessed by measuring the systolic displacement of the tricuspid annulus (TAPSE) and was measured from the apical plane of 4 cameras with the M-mode cursor aligned on the direction of the lateral tricuspid annulus, according to the current recommendations. By means of pulsed Doppler study of transmitral flow velocities in diastole, the filling flow of the LV was studied in the four-chamber apical view. All the studies were carried out by a same cardiologist in the same period of the clinical evaluation and the extraction of blood samples. Patients were classified according at their clinical involvement into affected [left maximal ventricular wall thickness ≥13 mm and/or abnormal ECG (with at least one of the following characteristics: PR < 120 or >200 ms, QRS > 120 ms, inverted T wave or history of significant arrhythmia)] or unaffected (i.e. non-affected) patients. From among the hospital staff and attendees of patients, two different sets of age- and sex- matched healthy controls were included, given the large gap in age and sex detected between affected and unaffected patients. Thus, affected patients were compared against one set of matched controls, and unaffected patients were compared against another set of matched controls.

The MSSI was used to evaluate the clinical involvement in FD patients and was calculated at inclusion. The MSSI scheme is composed of four sections covering general, neurological, cardiovascular and renal signs and symptoms of FD^8^. We used this tool to evaluate usefulness of biomarkers for clinical cardiac implication based on this accepted score in FD patients.

The study was carried out according to the principles of the Declaration of Helsinki and was approved by the Ethics Committee of the Instituto de Investigación Sanitaria y Biomédica de Alicante and the Ethics Committee of the Hospital Clínico Universitario Virgen de la Arrixaca. All included patients gave written informed consent to participation.

### Blood samples and laboratory assays

At inclusion, a >12 hours fasting venipuncture was performed in all patients. Plasma and serum fractions were obtained by centrifugation for 15 minutes at 3500 g. Aliquots were stored at −40 °C to allow batch analysis in a blinded fashion. We determined selected biomarkers of cardiac fibrosis (Galectin-3, Gal-3), cardiac wall stress (N-terminal proB-type natriuretic peptide, NT-proBNP), myocardial injury (high sensitivity troponin T, hsTnT), renal function (β-trace protein, BTP) and inflammation (interleukin-6, IL-6).

Gal-3 levels were determined on defrosted serum samples by ELFA (Enzyme-Linked Fluorescent Assay) in a Mini Vidas analyzer (Biomérieux®, France). The inter-assay and intra-assay coefficients of variation were 6.5% and 1.6%, respectively. The assay range was 3.3–100 ng/mL, with a lower limit of detection of 2.2 ng/mL and the limit of quantification at 3.3 ng/mL.

Serum levels of hsTnT were assayed by a Cobas® 6000 analyser (Roche Diagnostics, Mannheim, Germany). The inter-assay variation for hsTnT determining was 2.4%, with a lower detection limit of 3 ng/L.

Serum NT-proBNP was determined using a Roche Diagnostics proBNP assay on an Elecys 2010 analyzer (Roche Diagnostics, Mannhein, Germany) with an inter-assay variation of 3.5% and a detection limit of 5 pg/mL.

The determination of BTP was performed using a BN ProSpec analyzer (Dade Behring, Liederbach, Germany). The intra-assay and inter-assay coefficients of variation were 2.8% and 4.7%. Regarding renal function, it is important to note that the Modification of Diet in Renal Disease (MDRD-4) equation was used to estimate the Glomerular Filtration Rate (GFR) and the results were expressed in mL/min/1.73 m^2^.

Finally, concentrations of IL-6 were determined using Cobas® 6000 analyser (Roche Diagnostics, Mannheim, Germany). Within-run and total coefficients of variation of assays were <2% and 4.9%, respectively.

### Statistical analysis

First, we calculated sample size for testing biomarkers differences and a probability of a type-I error (alpha error) of 5% (0.05). The selected type-II error (β error) and the study power (Power = 1 − β) were 20% (0.2) and 80%, respectively. Our sample calculations demonstrated that a minimum of 19 subjects in each (patient or healthy control) arm was necessary.

Regarding the statistical analyses, categorical variables are presented as counts (percentages), while continuous variables are presented as mean ± SD (standard deviation) or median (interquartile range, IQR), as appropriate. The Kolmogorov-Smirnov test was used to check for normal distribution of continuous data.

Clinical variables were studied by bivariate correlations with the selected biomarkers. Receiver Operating Characteristic (ROC) curves were performed for evaluation of biomarkers levels related to patient’s organ involvement with the MSSI ≥ 20. Cut-off values were selected as the concentration of each biomarker with the best combination of sensitivity and specificity from ROC curves according to the Youden Index.

Univariate logistic regression analyses were performed to evaluate association between the selected cut-off of the biomarkers, as well as demographic or clinical variables, with cardiac involvement as assessed by the above indicated clinical parameters. Variables showing p-values < 0.15 where included into a multivariate regression model. Linear regression was used to show correlations between MSSI sub-scores and biomarker’s values. All p-values < 0.05 were accepted as statistically significant. Statistical analysis was performed using SPSS 19.0 for Windows (SPSS, Inc., Chicago, IL, USA).

## Results

We included 44 patients who were diagnosed with FD based on a molecular genetic analysis showing heterozygous or hemizygous mutation in the α-GAL-A-gene (GLA) as follows: 18 patients carrying mutation p.S238N (c.9091G > A), 10 showing mutation g.5052_5079del28, 4 with the mutation p.M187R, 4 patients with c.194 + 39delAT, 3 with p.W226C (c.226G > T) and p.R227X (c.227C > T), 2 patients carrying mutation p.Arg227x (c.679C > T), 2 showing mutation p.S126G (c.376A > G) and 1 patients with p.I253T(c.758T > C). Patients were classified attending their cardiac clinical phenotype into cardiac involvement (LVH or ECG abnormalities) or unaffected (without clinical manifestation or involvement). Table 1 summarizes baseline characteristics of both patients’ types. As expected, those affected patients were significantly older and predominantly males. Differences in ERT, New York Heart Association (NYHA) functional class, GFR, history of atrial fibrillation, LVH and left ventricular maximum wall thickness (LVMT), α-Galactosidase activity and Lyso-Gb3 levels or MSSI were present between the two cohorts (all < 0.05).

**Table 1:** Baseline characteristics.

	Affected patients (N = 21)	Unaffected patients (N = 23)	*p-value*
Age (years)	52.2 ± 11.4	38.4 ± 18.3	**0.004**
Male	15 (71.4)	6 (26.1)	**0.006**
ERT	16 (76.2)	5 (21.7)	**0.001**
α-Galactosidase activity	15.46 ± 9.95	47.12 ± 27.84	**0.001**
Lyso-Gb3 (mg/mL)	5.57 (4.40–9.24)	1.56 (0.76–3.12)	**0.003**
NYHA functional class ≥2	11 (52.4)	1 (4.3)	**0.002**
Previous MI	1 (4.8)	0 (0.0)	0.447
GFR	65.1 ± 47.5	112.0 ± 31.2	**0.001**
Albumin-to-creatinine ratio (mg/g)	14.3 (0.75–14.5)	4.0 (1.4–7.4)	**0.018**
Hypertension	11 (52.4)	5 (26.1)	0.121
Previous AF	5 (23.8)	0 (0.0)	**0.021**
LV hypertrophy	20 (95.2)	1 (4.3)	<**0.001**
ICD	3 (14.3)	0 (0.0)	0.100
LV maximum wall thickness	15.5 ± 6.1	11.4 ± 5.6	**0.025**
MSSI	18 (15–20.5)	3 (1–5)	**0.011**

AF = atrial fibrillation; ERT = enzyme replacement therapy; GFR = glomerular filtration rate (by the 4-variable Modification of Diet in Renal Disease [MDRD-4], equation); ICD = implantable cardioverter defibrillator; LV = left ventricular; MI = myocardial infarction; MSSI = Mainz Severity Score Index; NYHA = New York Heart Association.

### Concentration of biomarkers and correlation with clinical features

Table 2 shows biomarkers concentrations for each biomarker and cohort. All biomarkers were significantly increased in affected patients in comparison with unaffected patients. Similar results were obtained when analyzing only male patients (Supplementary Table 1). As expected, affected patients had also higher concentrations of all biomarkers compared to age and sex-matched healthy controls. Of note, we found that Gal-3, BTP and IL-6 levels were higher in unaffected patients when compared with age and sex-matched healthy controls (p = 0.018, p < 0.001 and p = 0.036, respectively) (Table 2).

**Table 2:** Comparative analysis for biomarkers values between affected and unaffected patients and their respective healthy controls.

	Affected patients (N = 21)	Unaffected patients (N = 23)	*p-value*	Healthy controls 1 (N = 46)	p-value*	Healthy controls 2 (N = 27)	p-value**
Gal-3	16.6 ± 6.3	11.2 ± 2.7	**0.004**	10.9 ± 2.5	**0.002**	9.6 ± 1.8	**0.018**
NT-proBNP	1056.0 (69.4–2922.5)	50.2 (28.3–90.1)	**<0.001**	33.8 (19.8–61.9)	**<0.001**	31.7 (19.6–54.9)	0.089
hsTnT	25.3 (12.3–62.1)	4.5 (3.3–7.0)	**<0.001**	5.9 (4.0–8.2)	**<0.001**	4.7 (3.6–7.6)	0.516
BTP	0.83 (0.64–1.2)	0.62 (0.56–0.68)	**0.001**	0.52 (0.48–0.57)	**<0.001**	0.51 (0.47–0.53)	**<0.001**
IL-6	1.5 (1.5–2.6)	1.5 (1.5–1.5)	**0.006**	1.5 (1.5–1.5)	**0.001**	1.5 (1.5–1.5)	**0.036**

^*^Compared to affected patients. ^**^Compared to unaffected patients.

Healthy controls 1 = age and sex-matched to affected patients; Healthy controls 2 = age and sex-matched to unaffected patients.

We evaluated clinical features and differences in conventional echocardiographic parameters were found between affected and unaffected patients (Supplementary Table 2). Regarding correlations of biomarkers with clinical features, all of them demonstrated positive significant results for NYHA, LVH and LVMT (Table 3). Additionally, indexed left ventricular mass (ILVM) correlated with Gal-3, NT-proBNP, IL-6 and BTP, whereas glomerular filtration rate showed a significant negative correlation with Gal-3, NT-proBNP, hsTnT and BTP. Left ventricular ejection fraction (LVEF) did not correlate with any of the biomarkers, and Tricuspid Annular Plane Systolic Excursion (TAPSE), as marker of right ventricular systolic function, only negatively correlated with Gal-3 levels. All measured biomarkers correlated each other, with the exception of IL-6 and BTP (Table 3).

**Table 3:** Correlations between biomarkers and clinical features.

Clinical Feature or Biomarker	Gal-3		NT-proBNP		hsTnT		IL-6		BTP	
	*r*	*p-value*	*r*	*p-value*	*r*	*p-value*	*r*	*p-value*	*r*	*p-value*
New York Heart Association	0.50	**0.001**	0.53	**<0.001**	0.55	**<0.001**	0.40	**0.010**	0.50	**0.001**
LV hypertrophy	0.53	**<0.001**	0.61	**<0.001**	0.75	**<0.001**	0.41	**0.008**	0.40	**0.010**
LV maximum wall thickness	0.32	**0.047**	0.49	**0.001**	0.61	**<0.001**	0.43	**0.006**	0.52	**0.001**
Indexed left ventricular mass*	0.86	**<0.001**	0.39	**0.019**	0.25	0.127	0.57	**<0.001**	0.51	**0.002**
LV ejection fraction**	−0.26	0.111	−0.09	0.588	−0.29	0.064	−0.06	0.705	−0.16	0.328
TAPSE	−0.50	**0.019**	−0.12	0.558	−0.21	0.318	−0.25	0.260	−0.13	0.558
Glomerular filtration rate	−0.57	**0.002**	−0.67	**<0.001**	−0.64	**<0.001**	−0.24	0.245	−0.44	**0.025**
Gal-3	—		—		—		—		—	
NT-proBNP	0.43	**0.004**	—		—		—		—	
hsTnT	0.75	**<0.001**	0.64	**<0.001**	—		—		—	
IL-6	0.38	**0.015**	0.52	**0.001**	0.40	**0.010**	—		—	
BTP	0.62	**<0.001**	0.33	**0.037**	0.54	**<0.001**	0.29	0.074	—	

LV = left ventricular; TAPSE = Tricuspid Annular Plane Systolic Excursion.

*The mass of the left ventricle in grams was calculated by the linear method from the diameters and ventricular thicknesses with the formula = 0.8 × {1.04 × [([left ventricular end-diastolic dimension + interventricular septal thickness at end-diastole + posterior wall thickness at end-diastole]^3^-left ventricular end-diastolic dimension^3^)]} + 0.6.

**Measured with Simpson biplane method (apical four and two cameras).

### Cut-off points associated with clinical cardiac involvement

In order to establish cut-off points associated with clinical cardiac involvement, we constructed ROC curves for biomarkers’ levels depending on the affectation measured as MSSI score >20 (Supplementary Fig. 1). When the analysis was performed for Gal-3 levels, we selected a cut-off point of 9.5 ng/mL, with a sensitivity value of 1.00 and a specificity value of 0.70. As for NT-proBNP levels, the selected cut-off point was 206.4 pg/mL, with a sensitivity and specificity values of 1.00 and 0.88, respectively. Regarding hsTnT, ROC analysis showed a cut-off point (sensitivity, specificity) of 12.42 pg/mL (1.00–0.82). For BTP, the best cut-off (sensitivity, specificity) value was 0.81 mg/L (0.86, 0.88). Finally, corresponding figures for IL-6 were 1.57 pg/mL (0.71, 0.85).

Using these cut-off points all biomarkers with the exception of Gal-3 were significantly associated with cardiac clinical involvement, as defined by or clinical criteria, on univariate analyses. In addition, age, male sex and GFR were also associated with clinical cardiac involvement. However, on multivariate analysis, only hsTnT (OR 30.69, 95% CI 2.70–348.42; p = 0.006) and male sex (OR 8.17, 95% CI 1.16–57.75; p = 0.035) remained independently associated (Table 4).

**Table 4:** Logistic regression analysis for cardiac affectation.

Biomarker* or Clinical Feature	Univariate Analysis	Multivariate Analysis (conditional mode)
	OR (95% CI); *p-value*	OR (95% CI); *p-value*
**Gal-3** (Cut-off point; Sensitivity-Specificity) 9.5 ng/mL; (1.00–0.70)	2.04 (0.44–9.34); 0.361	
**NT-proBNP** (Cut-off point; Sensitivity-Specificity) 206.4 pg/mL; (1.00–0.88)	40.86 (4.51–370.44); **0.001**	3.93 (0.19–81.60); 0.116
**hsTnT** (Cut-off point; Sensitivity-Specificity) 12.42 pg/mL; (1.00–0.82)	198.00 (16.58–2364.88); <**0.001**	30.69 (2.70–348.42); **0.006**
**BTP** (Cut-off point; Sensitivity-Specificity) 0.81 mg/L; (0.86–0.88)	24.75 (2.69–227.61); **0.005**	1.74 (0.11–27–24); 0.597
**IL-6** (Cut-off point; Sensitivity-Specificity) 1.57 pg/mL; (0.71–0.85)	9.33 (1.65–52.92); **0.012**	3.76 (0.13–106.42); 0.095
Age	1.06 (1.04–1.11); **0.011**	1.05(0.94–1.18); 0.179
Male sex	7.08 (1.88–26.72); **0.004**	8.17 (1.16–57.75); **0.035**
Glomerular filtration rate	10.3 (1.01–1.06); **0.005**	0.99 (0.95–1.05); 0.454

*Selected cut-off points and Sensitivity/1-Specificity parameters form ROC curves for clinical affectation (Mainz Severity Score Index ≥ 20).

CI = Confidence Interval; OR = Odds Ratio.

### Biomarkers for global and organ affectation with MSSI

**Figure 1:**
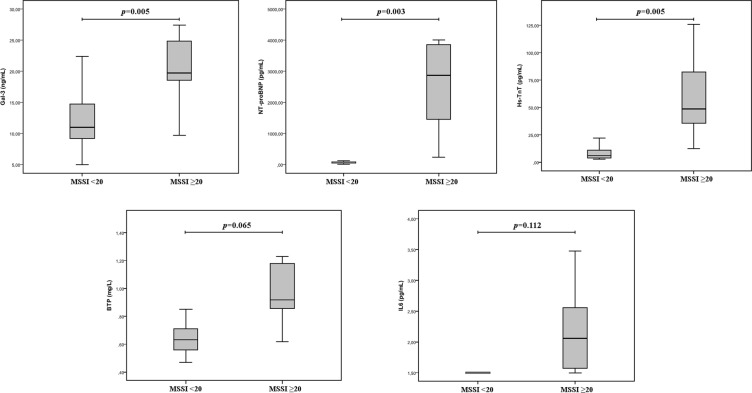
Concentration of biomarkers depending on Fabry disease patients’ global affectation (measured by MSSI).

As shown in Fig. 1, Gal-3, NT-proBNP and hsTnT levels were significantly higher in patients with MSSI ≥ 20 (p-values = 0.005; 0.003 and 0.005, respectively), whereas BTP showed a non-significant trend (p = 0.065) and IL-6 did not demonstrate to be significantly increased in patients with MSSI ≥ 20.

As it is already stated in Methods, the MSSI is composed of four sub-scores. Thus, Gal-3 levels were associated with cardiac and renal involvement sub-scores (both p < 0.001). On the other hand, NT-proBNP and hsTnT levels were associated with general (both p < 0.001), cardiac (p = 0.001 and p < 0.001, respectively) and renal (p = 0.014 and p = 0.001, respectively) subsections. Finally, BTP levels were associated with cardiac (p = 0.001) and renal sub-scores (p < 0.001) whereas IL-6 levels were only associated with cardiac sub-score (p = 0.011) (Table 5).

**Table 5:** Linear regression analysis for organ involvement.

MSSI sub-score	β coef. (95% CI)	*p-value*
**Gal-3**		
General	0.98 (−0.41–2.67)	0.162
Neurological	−0.23 (−0.79–0.33)	0.406
Cardiac	0.51 (0.32–0.70)	**<0.001**
Renal	0.58 (0.34–0.82)	**<0.001**
**NT-proBNP**		
General	823.82 (629.84–1037.81)	**<0.001**
Neurological	118.80 (−111.23–348.71)	0.303
Cardiac	170.82 (74.51–207.11)	**0.001**
Renal	161.73 (35.01–288.40)	**0.014**
**hsTnT**		
General	7.58 (3.99–11.17)	**<0.001**
Neurological	1.89 (−1.04–4.81)	0.200
Cardiac	3.05 (2.01–4.09)	**<0.001**
Renal	2.68 (1.15–4.21)	**0.001**
**BTP**		
General	0.05 (−0.08–0.18)	0.436
Neurological	−0.02 (−0.05–0.05)	0.934
Cardiac	0.04 (0.01–0.06)	**0.001**
Renal	0.06 (0.04–0.08)	**<0.001**
**IL-6**		
General	0.05 (−0.13–0.23)	0.575
Neurological	−0.03 (−0.10–0.04)	0.402
Cardiac	0.04 (0.01–0.07)	**0.011**
Renal	0.03 (−0.01–0.06)	0.196

CI = confidence interval; MSSI = Mainz Severity Score Index; OR = odds ratio.

## Discussion

FD is a multiorganic disease affecting different systems such as cardiovascular, neurologic or renal. In the present study we have evaluated biomarkers for cardiac fibrosis (Gal-3), cardiac wall stress (NT-proBNP), myocardial injury (hsTnT), renal function (BTP) or inflammation (IL-6) in patients with FD. In summary, we found differences in all the measured biomarkers between affected and non-affected patients.

One of our main results is that three measured biomarkers (Gal-3, BTP and IL-6) appeared higher in clinically non-affected patients than in matched controls, suggesting their potential usefulness for early detection of cardiac affectation. Until now, only clinical tools are available for evaluation of affectation signs in Fabry patients. We hereby demonstrate that the mentioned biomarkers are higher in patients showing no clinical involvement when compared with age and sex-matched controls. However, given that IL-6 showed the majority of values under the detection limit, it seems not to be the biomarker of choice for this purpose, despite its statistically significant differences.

In addition, when attending at the cardiac clinical affectation into the patients’ cohort, we found that all the analysed biomarkers were raised in the affected versus unaffected patients. Patients within the unaffected group were younger and predominantly female, as has been previously described^18–20^.

In our study, all biomarkers with the exception of IL-6 correlated with functional class (NYHA functional class), renal function (GFR) or cardiac hypertrophy (LVH or IVM). Gal-3 was the best biomarker for clinical cardiac involvement, and was significantly associated with the whole spectrum of the studied correlations. This biomarker has been proposed as biomarker of fibrosis or remodelling^21^, and it is higher in patients with lysosomal diseases^22^. We therefore propose that Gal-3 may be implicated in the early stages of organ involvement in patients with FD, including cardiac or renal remodelling, as suggested by the strong association with MSSI sub-scores and all the parameters of clinical involvement. This hypothesis is also supported by our observation that Gal-3, together with BTP, was significantly higher in the unaffected versus healthy control group. Hence, both biomarkers could be useful for early detection of sub-clinical affectation or for therapy follow-up.

Here, we have also proposed a cut-off point for each biomarker of cardiac clinical affectation. By applying our criteria and the accepted MSSI for FD patients, only hsTnT and male sex were independently associated in the multivariate analysis. Our group previously reported that hsTnT remains high in patients with stable hypertrophic cardiomyopathy (HCM), indicating that this biomarker may reflect a continuous myocyte loss associated with parameters of HCM severity^23^. Previous studies reported higher concentrations of TnI in FD compared with healthy controls, associated with LVH^24^. In addition, Seydelmann *et al*.^25^ found that increased hsTnT levels in FD patients with advanced cardiomyopathy were highly correlated with fibrosis detected on cardiac magnetic resonance imaging. Other groups, have demonstrated that also biomarkers of endothelial dysfunction such symmetric dimethylarginine (SDMA) and L-homoarginin/SDMA may be involved in Fabry related cardiomyopathy^26^.

Herein we also demonstrate that Gal-3 and BTP were associated with cardiac and renal impairment and they are higher in patients at early stages of organ involvement. In advanced affected patients, hsTnT emerges an independent risk factor for clinical cardiac involvement, together with male sex, with values of hsTnT ≥12.42 pg/mL as potential useful cut-off for the prediction of clinical cardiac involvement.

### Limitations

This study is limited mainly by its observational design so we could explore only associations, and no causality is implied. Although biomarkers’ levels resulted clearly higher in affected patients, we cannot ignore possible changes in their levels over time. In addition, we cannot exclude the involvement of other variables not included in the present study. Another limitation that must be acknowledged is the sample size, since FD is a rare, progressive disease. Larger cohorts, perhaps from multicentre international studies, are desirable to corroborate our results.

## Conclusion

In the present study we demonstrate that Gal-3 and BTP levels are significantly increased in clinically unaffected patients with FD in comparison with healthy controls. This finding suggests that these biomarkers could be useful for the early detection of cardiac affectation in patients with FD. On the other hand, hsTnT and male sex are independent risk factors for established clinical cardiac involvement in FD.

## Supplementary information

Supplementary Dataset 1
