# Supplementary material for: Galectin-3 and β-trace protein concentrations are higher in clinically unaffected patients with Fabry disease
Source: Sci Rep. 2019 Apr 17;9:6235. doi: 10.1038/s41598-019-42727-4 (PMC6470309; doi:10.1038/s41598-019-42727-4)

Galectin-3 and β-trace protein concentrations are higher in clinically unaffected patients with Fabry disease

Running head: Gal-3 and BTP in Fabry disease

Diana Hernández-Romero[1], Jessica Sánchez-Quiñones[2], Juan Antonio Vílchez[3], José Miguel Rivera-Caravaca[1], Gonzalo de la Morena[1], Gregory Y.H. Lip[4], Vicente Climent[2], Francisco Marín[1]

1. Department of Cardiology, Hospital Clínico Universitario Virgen de la Arrixaca, Instituto Murciano de Investigación Biosanitaria (IMIB-Arrixaca), University of Murcia, CIBERCV, Spain.
2. Department of Cardiology, Hospital General Universitario de Alicante, Alicante, Spain.
3. Department of Clinical Analysis, Hospital General Universitario Santa Lucía, Instituto Murciano de Investigación Biosanitaria (IMIB-Arrixaca), Cartagena, Spain.
4. Liverpool Centre for Cardiovascular Science, University of Liverpool and Liverpool Heart & Chest Hospital, Liverpool, United Kingdom and Aalborg Thrombosis Research Unit, Department of Clinical Medicine, Aalborg University, Aalborg, Denmark.

Corresponding author:

Diana Hernández-Romero, PhD

Department of Cardiology, IMIB-Arrixaca

Ctra. Madrid-Cartagena s/n, 30120 Murcia, Spain

Phone: +34 868888151; Fax: +34 868888115; Email address: dianahr@um.es

Supplementary Table 1. Comparative analysis for biomarkers values between affected and unaffected male patients.

Biomarker	Affected patient (N=15)	Unaffected patient (N=6)	p-value
Gal-3	18.5 (13.0-22.7)	9.5 (7.6-14.4)	**0.001**
NT-proBNP	369.7 (63.5-3327.0)	27.0 (14.7-39.7)	**<0.001**
hsTnT	26.4 (13.5-56.3)	6.1 (4.5-8.4)	**<0.001**
BTP	0.88 (0.70-1.30)	0.68 (0.60-0.74)	**0.015**

Supplementary Table 2. Comparative analysis for conventional echocardiographic parameters between affected and unaffected patients

	**Unaffected FD patients (N=23)**	**Affected FD patients (N=21)**	**p-value***
Septum (mm)	9.1 ± 1.7	15.7 ± 2.3	<0.001
Posterior wall (mm)	9.2 ± 1.4	14.9 ± 2.6	<0.001
Maximun thikness (mm)	9.6 (9.0-11.0)	16.4 (15.0-18.0)	<0.001
DDLV (mm)	43.0 (41.0-52.0)	42.5 (41.0-53.0)	0.766
SDLV (mm)	27.0 (24.0-32.0)	29.5 (26.0-36.0)	0.348
LV mass (g)	116.1 ± 39.9	280.5 ± 90.2	<0.001
Indexed left ventricular mass (g/m2)	63.8 ± 22.9	148.6 ± 48.1	<0.001
iTDV (ml/m2)	45.7 ± 8.5	54.2 ± 12.2	0.021
iTSV (ml/m2)	17.2± 7.0	20.0 ± 9.3	0.298
LVEF (%)	66.1 ± 6.5	62.2 ± 11.9	0.195
LA diameter (mm)	30.2 ± 4.7	38.4 ± 5.2	<0.001
TAPSE (mm)	24.3 ± 3.3	23.0 ± 3.7	0.413
E velocity (cm/s)	0.90 ± 0.20	0.70 ± 0.10	<0.001
A velocity (cm/s)	0.60 ± 0.20	0.70±0.20	0.442
E/A ratio	1.51 (1.21-2.07)	0.96 (0.89-1.29)	0.003
E/e' septal	10.70 ± 3.70	13.90 ± 4.10	0.042
E/e' lateral	8.60 ±3.20	11.30 ± 4.40	0.067
DTE (ms)	188.0 ± 37.8	229.6 ± 43.5	0.005

Echocardiographic parameters in the included FD groups. The magnitudes express the mean and standard deviation, or the median and interquartile range, as appropriate. * Level of statistical significance of the comparison between subgroups with and without cardiac involvement. A: maximum velocity of the end-diastolic wave of the transmitral filling flow; AI: left atrium; E: maximum velocity of the protodiastolic wave of the transmitral filling flow; ECG: electrocardiogram; DDLV: end-diastolic diameter of the left ventricle; SDLV: end-systolic diameter of the left ventricle; FD: Fabry disease; LVEF: left ventricular ejection fraction; LVH: left ventricular hypertrophy; I. Mass VI: mass index of the left ventricle; TAPSE: tricupid annulus plane systolic excursion; DTE: deceleration time of the E wave; VI: left ventricle; iTDV: indexed end-diastolic volume; iTSV: indexed telesystolic volume.

Supplementary Figure 1. Receiver operating characteristic curves of biomarkers levels related to patient's affectation (MSSI ≥20).

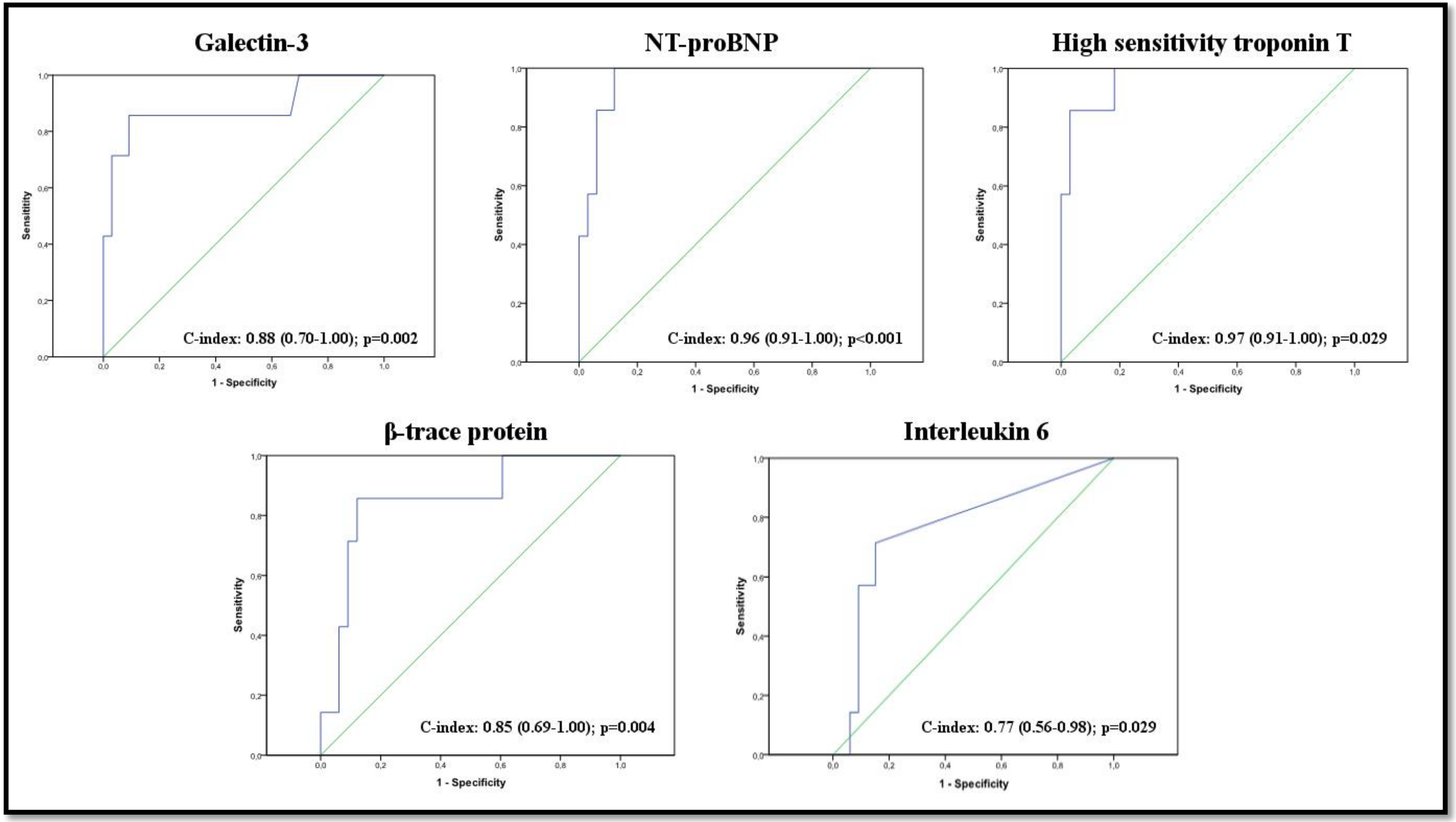

Supplement: Supplementary file 1 — Supplementary Dataset 1 [file 41598_2019_42727_MOESM1_ESM.pdf]
